# Copeptin analysis in endocrine disorders

**DOI:** 10.3389/fendo.2023.1230045

**Published:** 2023-10-04

**Authors:** Nareshni Moodley

**Affiliations:** ^1^ Department of Chemical Pathology, Inkosi Albert Luthuli Central Hospital, National Health Laboratory Services, Durban, South Africa; ^2^ Department of Laboratory Medicine (Chemical Pathology), University of Kwa-Zulu Natal, Durban, South Africa

**Keywords:** copeptin, diabetes insipidus, arginine vasopressin, endocrine, biomarker

## Abstract

Copeptin is cleaved from the same precursor as arginine vasopressin and is released in equimolar amounts with arginine vasopressin from the posterior pituitary in response to the same stimuli. Its level of stability in the blood, quick and simple analysis, and ease of automation make it much easier to analyze than arginine vasopressin, thereby offering a suitable alternative to measuring arginine vasopressin in endocrine disorders. Research has demonstrated the suitability of copeptin in adults for the differentiation of arginine vasopressin resistance and arginine vasopressin deficiency from primary polydipsia, in addition to the early identification of arginine vasopressin deficiency following pituitary surgery; however, further research is still required in the Syndrome of Inappropriate Antidiuretic Hormone (SIADH) and the pediatric population.

## 1 Introduction

Copeptin is the 39-amino acid glycopeptide C-terminal portion of the precursor peptides preprovasopressin and provasopressin, from which arginine vasopressin (AVP) is also cleaved for release. AVP plays a pivotal role in the endocrine stress response by stimulating adrenocorticotrophic hormone release and in osmotic and cardiovascular homeostasis by promoting water conservation in the body *via* the kidney. It is predominantly produced in the hypothalamus but also in other tissues like the sympathetic ganglia, adrenal glands, and testes. The short plasma half-life of AVP of 5-20 min, high instability in plasma even when frozen, and high degree of platelet binding (over 90%) requiring complete pre-analytical removal of platelets make AVP difficult to measure. Copeptin is stable in plasma, needs no special pre-analytical treatment, and can be easily measured using many assays with small sample volumes and results in as little as 0.5-2.5 hours (1–3). Studies have found that blood copeptin levels are similar to blood AVP levels, making copeptin a suitable alternative to AVP (4, 5). Balanescu et al. reported that copeptin concentrations correlated more closely with plasma osmolality than AVP (5). Copeptin has also been shown to be stable for at least 7 days at room temperature and 14 days at 4°C (3, 6).

## 2 What we know about copeptin, the molecule

### 2.1 Formation

**Figure 1:**
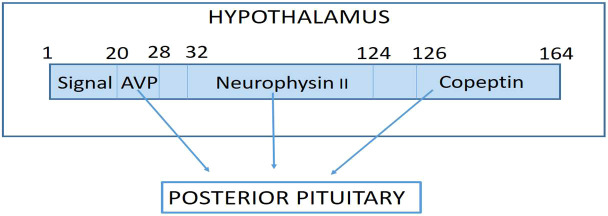
Pre-provasopressin is the precursor hormone of arginine Vasopressin (AVP) which is formed in the magnocellular nuclei of the hypothalamus, where the signal peptide is cleaved to form pro-AVP. Pro-AVP folds to place AVP in the binding pocket neurophysin II, so that it is protected from proteolysis. During transport to the posterior pituitary, AVP is cleaved off pro-AVP and finally another cleavage step seperates neurophysin II from copeptin with complete processing of the precursor hormones at the level of the posterior pituitary where all three molecules are stored and released into the blood in response to stimuli such as increased Osmolality, decreased blood pressure, decreased blood volume and/or stress.

The precursor peptides preprovasopressin and provasopressin are mainly produced in the magnocellular neurons of the hypothalamus and are enzymatically separated into the nine-amino acid peptide arginine vasopressin (AVP), 39 amino acid copeptin (CTproAVP), and neurophysin II, all of which are released into the blood in equimolar concentrations from the posterior lobe of the pituitary gland primarily in response to decreased blood volume, high blood osmolality, stress, and/or low blood pressure (**Figure 1**) (1, 2).

### 2.2 Physiological function

The physiological role of copeptin is not fully defined; however, studies have shown that it is a chaperone-like molecule for pro-AVP formation and that it monitors protein folding and interacts with many glycosylated proteins through interaction with the calnexin/calreticulin system, thus increasing the formation of active hormone and decreasing the production of inactive hormone (2).

### 2.3 Elimination

The elimination of copeptin is also not fully understood; however, it is eliminated at least in part by the kidneys, with an inverse relationship between copeptin blood levels and glomerular filtration rate in patients with chronic kidney disease (2).

### 2.4 Measurement

Numerous copeptin immunoassays and enzyme-linked immunosorbent (ELISA) assays have been developed. The two most studied immunoassays are an original sandwich immunoluminometric assay (LIA) and an automated immunofluorescent immunoassay (on the KRYPTOR platform), which show good correlation with very low and high levels of copeptin. However, the ELISA assay correlates poorly with both immunoassay methods, and therefore cut-offs developed on the immunoassay systems cannot be used for the ELISA assay results (2, 3).

## 3 Clinical applications in endocrinology

### 3.1 Diabetes insipidus

Diabetes insipidus (DI) is a condition characterized by disordered arginine vasopressin (AVP) secretion or action, resulting in the production of hypotonic urine (<300 mOsm/kg H2O) >50 ml/kg/day, with concurrent polydipsia (>3 L/day). It results from either decreased secretion of AVP (AVP deficiency) or resistance to AVP action (7).

AVP deficiency has various etiologies, such as pituitary or hypothalamic median eminence lesions, trauma, pituitary surgery, neoplastic, vascular, autoimmune, infectious, granulomatous, or hereditary forms (2, 7). Complete AVP resistance is due to a lack of aquaporin 2‐mediated water reabsorption in the renal collecting duct, which may be due to electrolyte disturbances (hypercalcemia or hypokalemia, renal pathologies, gene mutations in the key proteins vasopressin V2 receptor or aquaporin 2) or secondary to adverse drug effects (e.g., lithium) (2, 3).

Patients with primary polydipsia have chronic excessive fluid intake (which can occur in health-conscious people who want to drink large amounts of water, who have dependency disorders or reduced thirst thresholds, and in psychiatric patients) with subsequent excretion of hypotonic urine, hence presenting similarly to DI. It is important to differentiate between AVP resistance, AVP deficiency, and primary polydipsia as their treatment differs and incorrect management could have dire consequences (2, 3).

The water deprivation test is the diagnostic gold standard for differentiating DI from its main differential diagnosis, primary polydipsia. During this test, patients are not allowed to ingest any water for a maximum period of 17 hours or until blood sodium concentrations exceed 150 mmol/l or 3-5% of the patient’s initial body weight is lost, with measurements of urine excretion, urine osmolality, blood sodium, and blood osmolality during the water deprivation period. At the end of the water deprivation period, exogenous AVP is administered, and changes in urine osmolality are assessed for a period of time according to institutional protocols. Complete AVP deficiency is diagnosed based on the recommendations of Miller et al. for a urine osmolality that does not rise to >300 mOsm/kg during the water deprivation period with a >50% increase post-exogenous AVP administration. AVP-resistant patients have no increase in urine osmolality after exogenous AVP administration, and partially AVP-deficient patients have a urine osmolality between 300 and 800 mOsm/kg during water deprivation with a >9% increase after AVP injection; however, this test has only 70% diagnostic accuracy and was developed in a small cohort of 29 patients. Current methods utilized to assess post-operative DI (serum and urine sodium, and osmolality and fluid balance determination) have low sensitivity and specificity (<50%) (3, 8). Studies have reported that copeptin is useful in various clinical conditions, especially in the differential diagnosis of polyuria-polydipsia syndrome (2, 9, 10).

Research has shown that an unstimulated, random copeptin cut-off of >21.4 pmol/L can diagnose AVP resistance with 100% sensitivity and specificity for diagnosis. Differentiating AVP deficiency from primary polydipsia does require stimulation testing due to the similar baseline copeptin values in these two conditions. The copeptin-based hypertonic saline stimulation test and the arginine stimulation test have been shown to have high diagnostic accuracy of 97% and 93%, respectively, for DI. The copeptin-based hypertonic saline infusion test has a diagnostic accuracy of 95-96.5% for partial DI and is safe and better tolerated than the water deprivation test (2, 10). Arginine is a less effective stimulus than copeptin, and therefore has a lower diagnostic accuracy but requires less blood sodium monitoring and is better tolerated (2). Atila et al. also assessed the copeptin-based glucagon stimulation test and found that glucagon-stimulated copeptin in healthy participants, using a copeptin cut-off level of 4.6 pmol/l, had a sensitivity of 100% and a specificity of 90% to discriminate between AVP deficiency and primary polydipsia (11). It has also been shown that post-operative copeptin results in patients after pituitary surgery are much lower in those who develop AVP deficiency than in those who do not (2, 8, 12–14); therefore, it could greatly assist in the prompt diagnosis of AVP deficiency in these subjects.

### 3.2 Syndrome of inappropriate antidiuretic hormone

SIADH is characterized by inappropriately elevated plasma AVP levels, decreased blood osmolality, inappropriately high urine osmolality, and normal or increased blood volume. It is a common cause of euvolemic hyponatremia in hospitalized patients. Causes of SIADH include brain pathology (such as surgery, tumors, infection, prolonged seizures, psychiatric disease, and stress), non-CNS tumors, lung disease, and certain medications (anticonvulsants, antiparkinsonian drugs, antipsychotics, antipyretics, antidepressants, angiotensin-converting enzyme inhibitors, antineoplastic drugs, and first-generation sulfonylureas) (15).

SIADH is diagnosed when blood osmolality is <275 mOsm/kg, blood sodium is ≤130 mmol/l, urine osmolality is greater than blood osmolality, urine sodium is high (usually >40 to 60 mmol/l), and cardiac, hepatic, renal, thyroid or adrenal failure, effects of pituitary surgery, diuretic therapy, or medications known to stimulate AVP have been excluded (15).

One study found persistently high copeptin values (>38 pmol/l) in patients with lung cancer and SIADH; however, cancer patients have many other reasons for increased AVP secretion, such as comorbidities, medications, vomiting, nausea, dehydration, or stress, and there is still insufficient evidence to support this (2). Nevertheless, this is an area of interest for future studies involving copeptin.

There has been one study that showed that the ratio of copeptin to urinary sodium could help differentiate SIADH from conditions with decreased blood volume; however, copeptin alone was insufficient (3). More research is required in this area to confirm this.

## 4 Physiologic range and pathologic cut-offs

Plasma concentrations of copeptin show a wide range between 1 and 13.8 pmol/l, with a median concentration of 4.2 pmol/l in healthy, normal-osmotic volunteers. Men have slightly but significantly higher copeptin levels than women. Copeptin has not been shown to be influenced by age, circadian rhythm, food intake or phases of the menstrual cycle, but copeptin levels decrease with oral fluid intake as low as 200 to 300ml (2, 3). There were no gender differences in copeptin levels during hypertonic saline infusion tests (3).

A random copeptin value of 21.4 pmol/l was found to have a diagnostic accuracy of 100% for AVP resistance (9). Another study also found random copeptin levels >20 pmol/l as good cut-offs for AVP resistance, with post-overnight water deprivation levels of <2.6 pmol/l indicating AVP deficiency (diagnostic accuracy of 78%), and a ratio of Δplasma copeptin levels before and after water deprivation to plasma sodium post water deprivation had a high diagnostic accuracy of 94% for AVP deficiency (3).

Stimulated copeptin values for differentiating AVP deficiency from primary polydipsia depend on the type of stimulation test performed. A cut-off of ≤4.9 pmol/l post-hypertonic saline infusion test has been suggested to diagnose AVP deficiency with high diagnostic accuracy of 96% (3, 9, 10). An arginine infusion test cut-off of ≤3.8 pmol/l has been used to diagnose AVP deficiency (2), and the possible cut-off for the glucagon stimulation test was found to be 4.6 pmol/l (11).

Initially, an insulin tolerance test was used to induce hypoglycemia in patients 3 months after transsphenoidal pituitary surgery, which showed low copeptin levels in patients with AVP deficiency of 3.7 ± 0.7 pmol/l, with hypoglycemic copeptin levels of <4.75 pmol/l having the best diagnostic accuracy of 100%. The surgery itself is a stressful event that can trigger AVP release; therefore, unstimulated post-surgery cut-offs were assessed. Suggested post-pituitary surgery cut-offs on day 1 post-op are <2.5 pmol/l for AVP deficiency and >30 pmol/l indicate no AVP deficiency (3, 8) or <3.6 pmol/l for AVP deficiency (14), whereas one study by Jang et al. found that day 2 copeptin values <3.1 pmol/l showed the best performance in predicting permanent AVP deficiency (13). Kim et al. found that 3 months after transsphenoidal pituitary surgery, copeptin values of <1.9 pmol/l with normal serum sodium results were the best cut-off value for permanent AVP deficiency with a diagnostic accuracy of 81.8%; however, a copeptin value of ≥3.5 pmol/l excluded AVP deficiency with a negative predictive value of 100% (16). The cut-off values are summarized in **Table 1**.

**Table 1:** Published copeptin cut-off values.

Clinical Scenario	Cut-off (pmol/l)
AVP resistance	>21,4 (9)
Post-overnight water deprivation test	<2,6 indicative of AVP deficiency (3)
Hypertonic saline infusion test	≤4,9 (3, 9, 10)
Arginine infusion test	≤3,8 (2)
Glucagon stimulation test	<4,6 (11)
Post-pituitary surgery - Day 1 - Day 2 - 3 months - 3 months + insulin tolerance test	- <2,5= AVP deficiency; >30= no AVP deficiency (3, 8) - <3,1= AVP deficiency (13) - <1,9+normal serum sodium= AVP deficiency; ≥3,5= no AVP deficiency (16) - <4,75 (3)

## 5 Discussion

In the field of endocrinology, copeptin has been shown to play an important role in differentiating DI from primary polydipsia (2, 9, 10) and there are promising results regarding its utility in the diagnosis of post-pituitary surgery DI (2, 8, 12–14). The determination of appropriate cut-offs in a larger cohort and different populations is still needed. Another area that requires more research is the utility of copeptin in SIADH with other biomarkers. There are very few studies in the pediatric population, which is an important area to highlight in future studies on the utility of copeptin in endocrinology.

## Author contributions

The author confirms being the sole contributor of this work and has approved it for publication.
